# Whole Y-chromosome sequences reveal an extremely recent origin of the most common North African paternal lineage E-M183 (M81)

**DOI:** 10.1038/s41598-017-16271-y

**Published:** 2017-11-21

**Authors:** Neus Solé-Morata, Carla García-Fernández, Vadim Urasin, Asmahan Bekada, Karima Fadhlaoui-Zid, Pierre Zalloua, David Comas, Francesc Calafell

**Affiliations:** 1 Institute of Evolutionary Biology (CSIC-UPF), Departament de Ciències Experimentals i de la Salut, Universitat Pompeu Fabra, Barcelona, Spain; 2 YFull–Research Group, Moscow, Russia; 3 Département de Biotechnologie, Faculté des Sciences de la Nature et de la Vie, Université Oran 1 (Ahmad Ben Bella), Oran, Algeria; 4 Laboratoire de Génetique, Immunologie et Pathologies Humaines, Faculté des Sciences de Tunis, Campus Universitaire El Manar II, Université El Manar, Tunis, Tunisia; 5 The Lebanese American University, Chouran, Beirut, Lebanon

## Abstract

E-M183 (E-M81) is the most frequent paternal lineage in North Africa and thus it must be considered to explore past historical and demographical processes. Here, by using whole Y chromosome sequences from 32 North African individuals, we have identified five new branches within E-M183. The validation of these variants in more than 200 North African samples, from which we also have information of 13 Y-STRs, has revealed a strong resemblance among E-M183 Y-STR haplotypes that pointed to a rapid expansion of this haplogroup. Moreover, for the first time, by using both SNP and STR data, we have provided updated estimates of the times-to-the-most-recent-common-ancestor (TMRCA) for E-M183, which evidenced an extremely recent origin of this haplogroup (2,000–3,000 ya). Our results also showed a lack of population structure within the E-M183 branch, which could be explained by the recent and rapid expansion of this haplogroup. In spite of a reduction in STR heterozygosity towards the West, which would point to an origin in the Near East, ancient DNA evidence together with our TMRCA estimates point to a local origin of E-M183 in NW Africa.

## Introduction

The male-specific region of the Y chromosome (MSY) is one of the workhorses of human population genetics. The avoidance of recombination of this region implies that haplotypes are passed almost intact from generation to generation. As a result, variation is introduced into this region only by mutation^1^. Single nucleotide polymorphisms (SNPs) and short tandem repeats (STRs) are the most commonly used type of variation when working with the Y chromosome. Because of their low mutation rate (10^−9^ substitutions/site/year)^2^, SNPs (and some short indels) tend to be unique events in human evolution and can be easily combined into haplotypes, known as haplogroups. These haplogroups have been used to build consistent phylogenies that show a particular ethno-geographical distribution^3–5^.

Until recently, the main problem with SNPs was their ascertainment bias produced by the narrow range of populations (or even of individuals) assessed for variation. Now, the advent of next generation sequencing (NGS) of the MSY has solved the problem of ascertainment bias and has allowed the systematic discovery of thousands of new SNPs from worldwide populations. Several studies^6–13^ have provided refined Y chromosome phylogenies in which branch lengths are proportional to time, permitting the direct estimation of the time to most recent common ancestor (TMRCA) of nodes, and the coalescence of their branches can be used to trace effective population size back in time. Despite the improvements of NGS, further work is needed to better understand the internal diversity of specific haplogroups. On the other hand, STRs are also commonly used in population genetic studies; because of their higher mutation rate (10^−3^–10^−4^)^14^, Y-STRs exhibit a higher degree of variation and are thus an optimal tool for discriminating between closely related Y chromosomes. Although their mutation processes can be rather complex and can saturate much faster than SNPs, STRs can also provide good time estimates for relatively recent events^1^.

Here, by using whole Y chromosome sequences, we intend to shed some light on the historical and demographic processes that modelled the genetic landscape of North Africa. Previous studies suggested that the strategic location of North Africa, separated from Europe by the Mediterranean Sea, from the rest of the African continent by the Sahara Desert and limited to the East by the Arabian Peninsula, has shaped the genetic complexity of current North Africans^15–17^. Early modern humans arrived in North Africa 190–140 kya (thousand years ago)^18^, and several cultures settled in the area before the Holocene. In fact, a previous study by Henn *et al*.^19^ identified a gradient of likely autochthonous North African ancestry, probably derived from an ancient “back-to-Africa” gene flow prior to the Holocene (12 kya). In historic times, North Africa has been populated successively by different groups, including Phoenicians, Romans, Vandals and Byzantines. The most important human settlement in North Africa was conducted by the Arabs by the end of the 7th century. Recent studies have demonstrated the complexity of human migrations in the area, resulting from an amalgam of ancestral components in North African groups^15,20^.

Besides geography, cultural diversity must also be considered. Two branches of languages belonging to the Afro-Asiatic family define two major groups in North Africa: Arabs and Berbers. Arabic languages and culture, as well as the Islamic religion, were brought from the Near East during the Islamic expansion. The Berber people, characterized for speaking Berber languages, are considered the direct descendants of the ancestral pre-Arabic peoples of North Africa^20^. However, the Berber language and ethnicity should not be equated: many Berber speakers live in large cities, particularly in Morocco and Algeria, and some populations with traditional lifestyles, such as the Reguibates, speak Arabic dialects. In spite of their cultural differences, Y-chromosome SNPs and STRs^21^, and autosomal haplotype-based methods^20^ have demonstrated the absence of strong genetic differences between Berbers and Arabs.

Studies based on the Y chromosome have highlighted E-M78 and E-M81 as the most frequent paternal lineages in North Africa, although they showed different distribution patterns. Whereas the frequency of E-M78 declines towards Northwest Africa, E-M81 has been found at high frequencies (71%) in Northwestern Africa and its frequency decreases towards the East; it is found sporadically in S Europe and E Africa, and it is practically absent elsewhere. These evidences suggest that E-M81 must be considered to explore the historical and demographical processes that gave rise to current North African populations. However, little is known about the phylogeographic structure of this haplogroup and its origin and emergence are still very controversial. While some studies pointed to a Palaeolithic origin^21^, other authors claimed that E-M81 may have a Neolithic origin^22^. The most likely scenario, as suggested by Fadhlaoui-Zid *et al*.^17^, is that the origin of E-M81 is more recent than previously reported.

In the present project, we analyse whole Y chromosome sequences from 32 North African individuals selected by carrying the derived allele at M183. M183 was first described by Karafet *et al*.^5^, and appears to be an extremely dominant subclade within E-M81, to the point that E-M81*(xM183) individuals are very rare. Since we found no samples derived for E-M81 and ancestral for E-M183, we selected our individuals on the basis of E-M183. The aim of the present study is to provide a phylogeographic refinement of this paternal lineage in order to shed light on the human population history of North Africa. By using whole Y chromosome sequences, we have been able to describe E-M183 subbranches that will be used to define whether this lineage presents any geographical substructure. Next, by using STRs we will interrogate the genetic diversity within E-M183 subclades in a larger dataset. Finally, both SNP data and STRs will be used to provide updated time constraints of the spread of E-M183.

## Results

In the present study, we analysed whole Y chromosome sequences from 32 North African males who belong to the most frequent haplogroup in North Africa: E-M81 (E-M183). Using the samples genotyped for the present study we constructed an updated map showing the paternal lineage distribution in North African populations compared to neighbouring European and Near Eastern populations (Supplementary Fig. S1). And as previously reported by Fadhlaoui-Zid^17^, E-M81 is predominant in Northwestern Africa and almost absent elsewhere.

**Figure 1:**
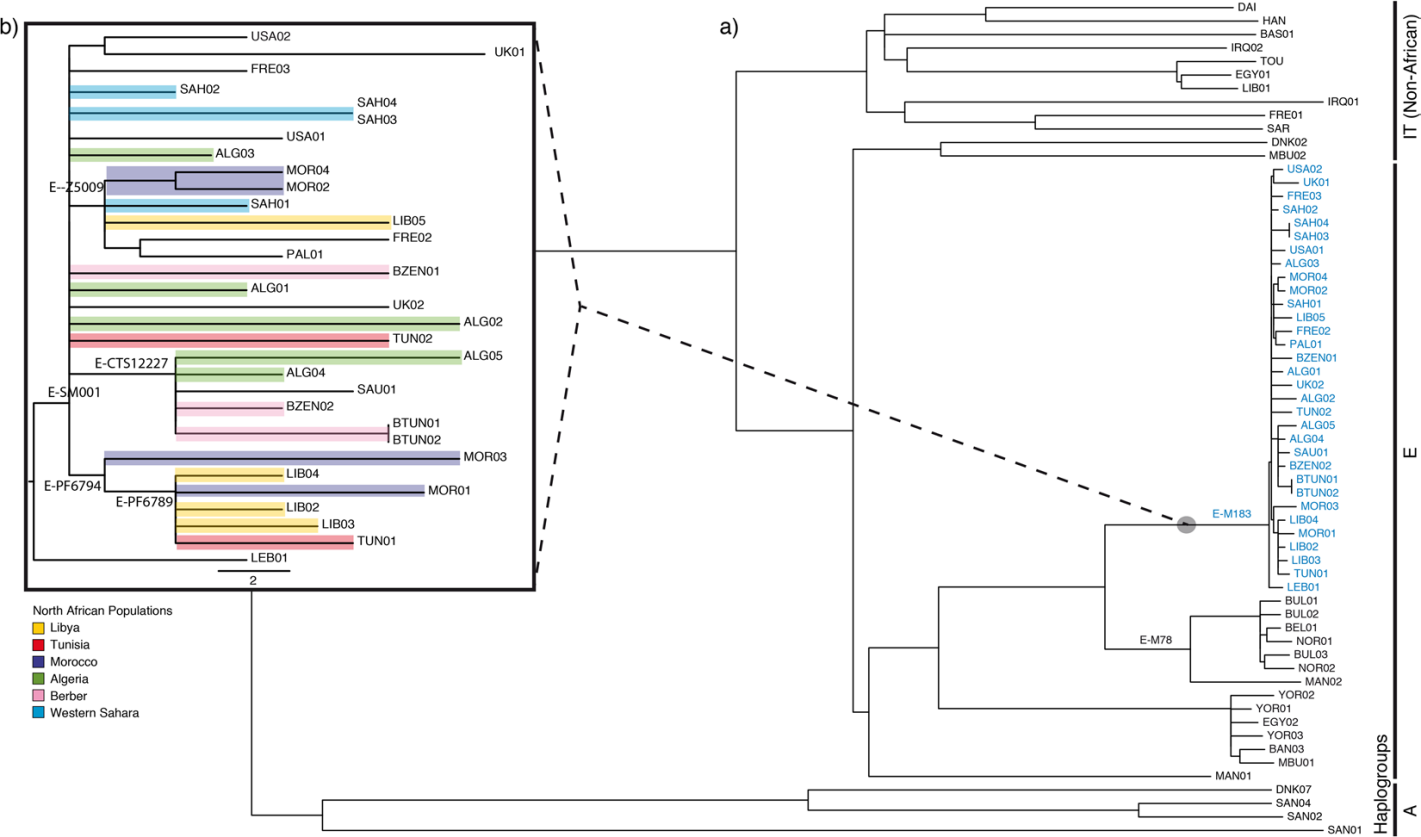
Maximum parsimony tree. Phylogenetic relations among (**a**) all samples included in the project (in blue the E-M183 branch) and (**b**) individuals within E-M183 branch are shown, coloured by its North African origin. Branch lengths are proportional to the number of SNPs on the branch.

The phylogenetic relationships among the E-M183 carriers and other African and non-African lineages are shown in Fig. 1a (see also Supplementary Table S1). The tree was consistent with the previous independent haplogroup assignation and the known phylogeny of the Y chromosome^5,7^. Moreover, the analysis of whole Y chromosome data enabled the characterization of five new subclades within this Y chromosomal branch (Fig. 1b). Although four of these five SNPs had previously been described, to the best of our knowledge this is the first academic publication describing their phylogenetic relationships and population frequencies. The first variant, named SM001, clusters together all E-M183 samples but one, a sample from Lebanon. Another subclade is defined by Z5009, which groups two Moroccan samples, and one sample each from the Western Sahara, Libya, Palestine and France. CTS12227 is shared by three Berber samples (two Tunisian Berbers and one Zenata Berber), two Arab Algerians and a sample from Saudi Arabia. Finally, three Libyan samples, two Moroccan and one Tunisian are derived for PF6794, and all but one of these samples are also derived for PF6789.

In order to study the geographical structure and phylogenetic robustness of E-M183, we genotyped the five subclades described using whole Y chromosome data in 250 North African samples (see methods). We also analysed the genetic diversity within and between North African populations by genotyping the set of Y-STRs contained in the AmpFlSTR®YFiler® kit.

### Differences between and within populations

**Figure 2:**
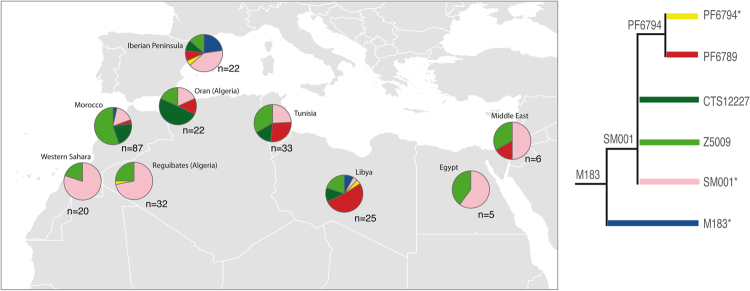
Distribution of E-M183 subclades among North Africa, the Near East and the Iberian Peninsula. Pie chart sectors areas are proportional to haplogroup frequency and are coloured according to haplogroup in the schematic tree to the right. n: sample size. Map was generated using R software^65^.

The distribution of each subhaplogroup within E-M183 can be observed in Table 1 and Fig. 2. Indeed, different populations present different subhaplogroup compositions. For example, whereas in Morocco almost all subhaplogorups are present, Western Sahara shows a very homogeneous pattern with only E-SM001 and E-Z5009 being represented. A similar picture to that of Western Sahara is shown by the Reguibates from Algeria, which contrast sharply with the Algerians from Oran, which showed a high diversity of haplogroups. It is also worth to notice that a slightly different pattern could be appreciated in coastal populations when compared with more inland territories (Western Sahara, Algerian Reguibates). And indeed, when we tested by AMOVA based on Y-STRs whether the coastal populations are different to more inland areas, we observe that 16.5% (P < 10^−5^) of the variance can be explained by differences among these two groups. Finally, surprisingly, Iberian samples showed the highest proportion of E-M183*, with a frequency over E-M183 chromosomes of 20%, whereas in North Africa the frequencies of M183* range from 0 to 7%. However, note that if these frequencies were given over all individuals (and not only over those carrying E-M183), then E-M183* would represent just 0.5% of all Iberian Y chromosomes, but it reaches 7.7% in Libyans.

**Table 1:** M183 absolute haplogroup frequencies by population.

Population	M183*	SM001*	PF6794*	PF6789	CTS12227	Z5009	Other	N	% E-M183
Western Sahara	0	16	0	0	0	4	6	26	76.92
Morocco	3	14	0	3	19	48	53	140	62.14
Algeria (Oran)	0	4	0	3	11	4	29	51	43
Algeria (Reguibates)	0	23	1	0	0	8	7	39	82.05
Tunisia	0	8	0	9	5	11	58	91	36.26
Lybia	2	1	1	13	3	5	51	76	32.89
Egypt	0	3	0	0	0	2	95	100	5
Near East	0	3	0	1	0	2	368	374	1.60
Iberian Peninsula	5	9	1	2	2	3	1062	1084	2.03

N: Sample size.

**Figure 3:**
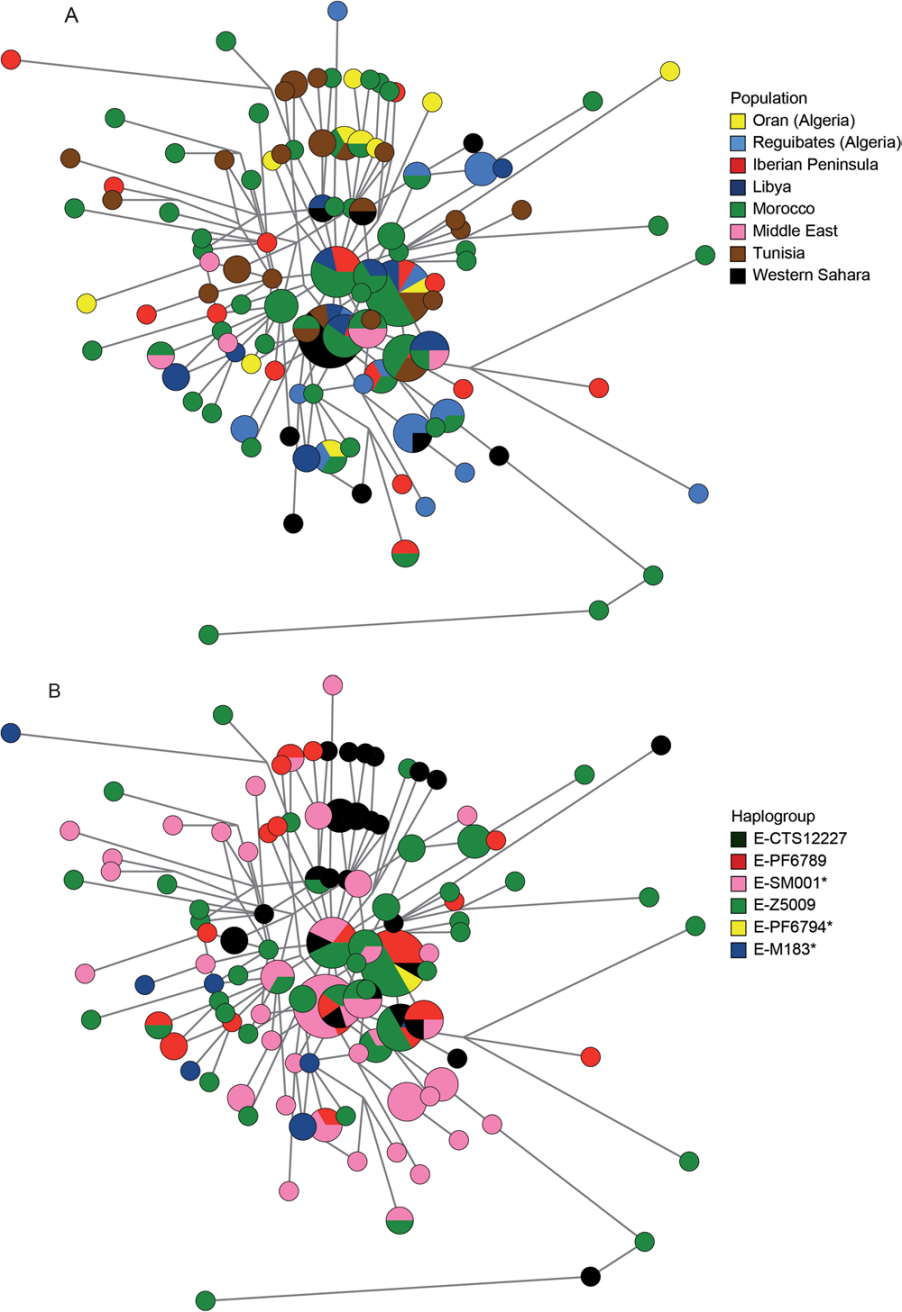
Median-joining network based on 13 Y-STRs: DYS19, DYS389I, DYS390, DYS391, DYS393, DYS438, DYS439, DYS437, DYS448, DYS456, DYS458, Y GATA H4, and DYS635. Each circle represents a haplotype with its size proportional to frequency and has been coloured according to (**a**) populations and (**b**) haplogroups (see key). The lines between them indicate the Y-STR mutational steps.

**Figure 4:**
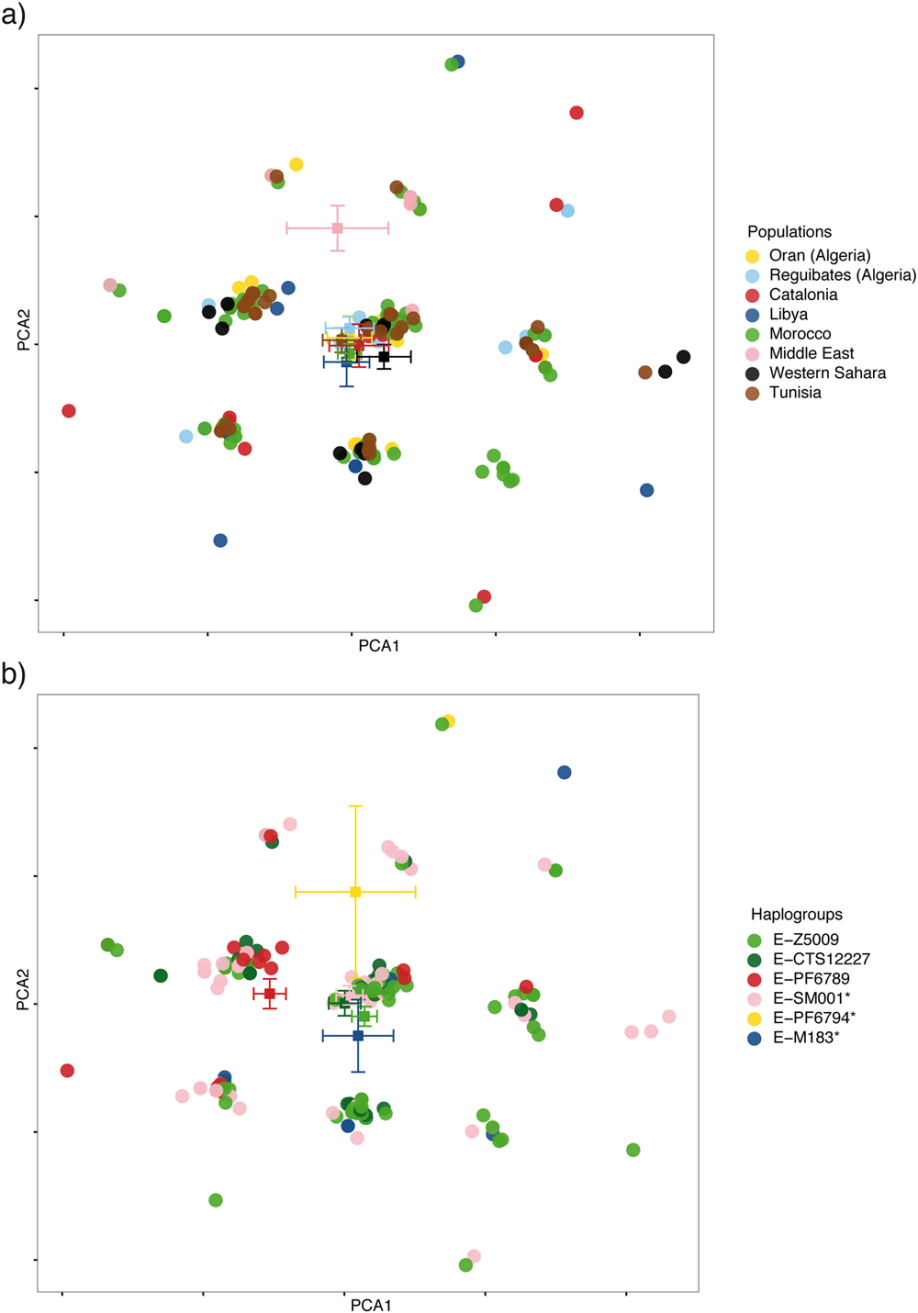
Principal component analysis based on Y-STRs. Coloured by (**a**) population and (**b**) by haplogroup (see key). Squares represent mean values of each component in the PCA coloured by (**a**) population and (**b**) haplogroup.

Median-joining networks based on Y-STR haplotypes data and coloured by population were used to investigate whether a particular geographical structure could be defined within this Y chromosomal lineage. As shown in Fig. 3a, apparently, no cluster by population can be distinguished. To better investigate the differences among populations we performed an AMOVA analysis based on Y-STRs. Our results showed that 8.1% (P < 10^−5^) of the overall genetic variation was explained by differences among populations. As a reference, *F*_*ST*_ based on Y-STRs among a similar set of N. African populations, irrespectively of haplogroup, was 11.2%^17^, suggesting that paternal structure in North Africa does not seem to correlate with population ascription. Moreover, principal component analysis based on Y-STRs and coloured by population (Fig. 4a) showed that no geographical cluster can be distinguished within the PCA. However, when we plotted the mean values of each component in the PCA, we observed that all populations clustered together except those from West Asia, and apparently the second component was driving this split (Fig. 4a and Supplementary Fig. S2b). Differences in average PC2 value were marginally statistically significant (ANOVA, P = 0.042, Supplementary Table S2). Taken together, our results pointed that little or no population-based structure can be found within E-M183.

### Age estimates

We have estimated the divergence of the E-M183 branch from its sister, E-M78, around 9,700 ya (Table 2) when using a fast mutation rate and ~12,700 ya when a slow mutation rate is considered (see methods). Both a frequentist (ρ) and a Bayesian method gave similar results. The TMRCA (Time to Most Recent Common Ancestor) of a certain haplogroup can provide some constraints on the time of its spread. Here, we used both Y chromosome SNP and STR data to obtain those estimates. Table 2 shows the TMRCA obtained for E-M183 and its sister clade (E-M78) by using SNP data from whole Y chromosome sequences, as well as the coalescence time for E-M183 and its subclades computed with Y-STR data. Regardless of using a Bayesian or a Rho-based approach, our findings when using SNP data suggest that E-M183 originated around 2,000 years ago (ya). It is worth to notice that when the tree is calibrated with a slow mutation rate^23,24^, the TMRCA of E-M183 given by BEAST reaches ~3,000 ya. However, age estimates computed using STR data strongly support that the coalescence time for this haplogroup is around 2,000 ya. We have also computed the coalescence times for each subclade by using Y-STRs (Table 2). The TMRCAs of E-SM001, E-CTS1227, and E-Z5009 are all ~2,000 ya and their confidence intervals broadly overlap with each other and with that of the whole of E-M183, pointing to a rapid radiation. On the contrary, E-PF6794 and its subclade E-PF6789 appear to be more recent, at ~1,500 ya. Interestingly, E-PF6789 is present in most of North Africa and the Near East (Table 1); if, as discussed below, E-M183 may have expanded from East to West, then ~1,500 ya sets an upper limit for this expansion.

**Table 2:** TMRCA estimates using a) SNP and b) Y-STR data.

(a)	TMRCA (Y-SNP data)					
	Bayesian approach		Rho-based approach			
Mutation rate	E-M78	E-M183	E-M78	E-M183		
10^−9^ substitutions/site/year^2^	9693 [8093, 11352]	2284 [1809, 2783]	9723 [8655, 10791]	1730 [1481, 1979]		
6.17 × 10^−10^ substitutions/site/year^24^	12675 [10572, 14837]	2984 [2365, 3639]	12758 [11356, 14160]	2270 [1943, 2597]		
**(b)**	**TMRCA (Y-STRs data)**					
	**Rho-based approach**					
**Mutation rate**	**E-M183**	**E-SM001**	**E-PF6794**	**E-M6789**	**E-CTS12227**	**E-Z5009**
1/858 years (www.yhrd.org, accessed on Dec. 14th, 2016)	2012 [1583, 2441]	1997 [1566, 2428]	1539 [1173, 1905]	1401 [1047, 1755]	2288 [1690, 2886]	1873 [1463, 2283]

Columns labelled *E-M78* refer to the divergence time between E-M78 and E-M183 rather than to the TMRCA of E-M78.

### Origin and dispersion

**Figure 5:**
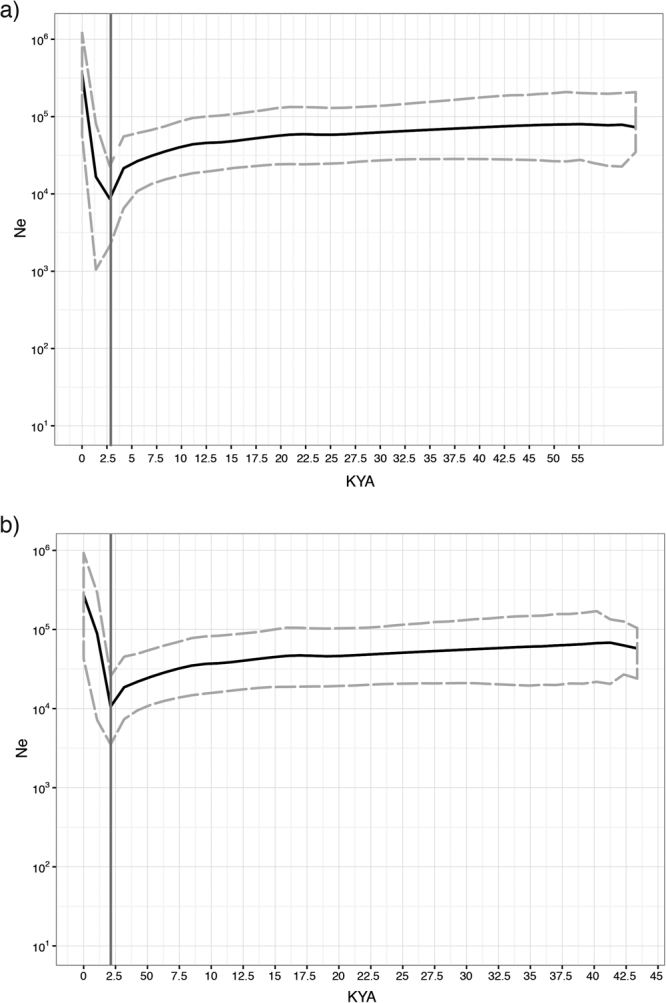
Bayesian skyline plots. Two different mutation rates have been used: (**a**) a ‘slow’ rate^24^ of 6.17 × 10^−10^ substitutions/site/year and (**b**) a ‘fast’ rate^2^ of 10^−9^ substitutions/site/ year. Black lines indicate the median effective population size (Ne) and discontinuous grey lines the 95% higher posterior density intervals. Vertical grey line indicate the TMRCA estimated using each substitution rate.

The star-like structure observed in the median-joining network of E-M183 (Fig. 3), could shed some light on the dispersion of E-M183. We found that Y-STRs are extremely homogeneous across E-M183 subhaplogroups, with the same haplotype shared by samples belonging to different subclades (Fig. 3b). This extreme homogeneity could be attributed to a recent and rapid radiation of this Y chromosomal branch^25,26^, which is also seen in the fact that most of its subclades seem to have appeared almost simultaneously. An AMOVA analysis showed that 8.6% (P < 10^−5^) of the genetic diversity is explained by differences among subclades, while the same type of analysis across haplogroups^27^ yields much higher values. Furthermore, in a principal component analysis based on Y-STRs and coloured by subclade (Fig. 4b) apparently no haplogroup cluster could be observed. However, the mean value for the first PC for E-PF6789 has a different sign than the rest, and the overall differences are statistically significant (ANOVA, P = 0.0049). In addition, despite the homogeneity of Y-STRs across E-M183 subhaplogroups mentioned above, we found a particular allele of DYS458 associated with PF6789. The frequency of allele DYS458*17 in PF6789-derived individuals is 71%, while it is much rare in other E-M183 chromosomes (31%) where allele DYS458*18 predominates (50%). When we turned to genetic diversity of each haplogroup (Supplementary Table S3), no significant differences are observed when comparing gene diversity and heterozygosity values between the different subhaplogroups, which again, could be due to a recent expansion of E-M183. However, PF6789 shows the lowest STR-based variance values. Next, Bayesian skyline analysis based on sequence data are in agreement with a recent expansion of this haplogroup. As shown in Fig. 5, regardless of using a) *slow* ^24^ or b) *fast* ^2^ mutation rates, we see a slight reduction and a rapid increase of population effective size around 2,000 ya. Finally, despite any geographical structure seems to be present within this North African Y chromosomal branch, other studies have proposed a correlation between the longitude and genetic diversity. To further investigate this correlation, we plotted the variance, the average gene diversity and the heterozygosity against the longitude of each population (Supplementary Fig. S3). Although a slight correlation has been observed between longitude and gene diversity (Spearman’s ρ = 0.77), as well as between longitude and STR allele size variance (ρ = 0.6), they are not statistically significant. When we turned to STR heterozygosity, the correlation (ρ = 0.89) becomes slightly significant (P = 0.03). However, this pattern appears to be driven by the fact that Western Sahara is the least diverse but most western population in our set.

## Discussion

Several studies have explored the paternal structure of North Africa showing that E-M183 is the most frequent paternal lineage in North Africa^17,22,28^. However, these analyses focused on targeted SNPs of the Y chromosome, preventing the discovery of new variation within its sequence. Here, by using whole Y chromosome sequences, we have been able to increase the knowledge of internal new branches within E-M183, which has led to a refinement of the phylogeography of this lineage, and to shed light on the controversial dates for its origin.

Our results evidenced that Y-STR haplotypes within E-M183 individuals are strikingly similar to each other and thus, subhaplogroups within E-M183 cannot be distinguished from each other based on Y-STR differences. As proposed by Larmuseau *et al*.^25^, the scenario that better explains Y-STR haplotype similarity within a particular haplogroup is a recent and rapid radiation of subhaplogroups. Although the dating of this lineage has been controversial, with dates proposed ranging from Paleolithic to Neolithic and to more recent times^17,22,28^, our results suggested that the origin of E-M183 is much more recent than was previously thought. Whereas other studies have relied only on STR data to provide time estimates, here, for the first time, we have used Y chromosomal sequence data to calculate the TMRCA for E-M183. As a result, we have been able to update the TMRCA for this haplogroup by using both SNP and STR data, avoiding a possible bias introduced by inferring the TMRCA using only rapid mutation rates. In addition to the recent radiation suggested by the high haplotype resemblance, the pattern showed by E-M183 imply that subhaplogroups originated within a relatively short time period, in a burst similar to those happening in many Y-chromosome haplogroups^23^.

Regarding the geographical origin of E-M183, a previous study^22^ suggested that an expansion from the Near East could explain the observed east-west cline of genetic variation that extends into the Near East. Indeed, our results also showed a reduction in STR heterozygosity towards the West (Supplementary Fig. S3), which may be taken to support the hypothesis of an expansion from the Near East. In addition, previous studies based on genome-wide SNPs^15,20^ reported that a North African autochthonous component increase towards the West whereas the Near Eastern decreases towards the same direction, which again support an expansion from the Near East. However, our correlations should be taken carefully because our analysis includes only six locations on the longitudinal axis, none from the Near East. As a result, we do not have sufficient statistical power to confirm a Near Eastern origin. In addition, rather than showing a west-to-east cline of genetic diversity, the overall picture shown by this correlation analysis evidences just low genetic diversity in Western Sahara, which indeed could be also caused by the small sample size (n = 26) in this region. Alternatively, given the high frequency of E-M183 in the Maghreb, a local origin of E-M183 in NW Africa could be envisaged, which would fit the clear pattern of longitudinal isolation by distance reported in genome-wide studies^15,20^. Moreover, the presence of autochthonous North African E-M81 lineages in the indigenous population of the Canary Islands, strongly points to North Africa as the most probable origin of the Guanche ancestors^29^. This, together with the fact that the oldest indigenous inviduals have been dated 2210 ± 60 ya, supports a local origin of E-M183 in NW Africa. Within this scenario, it is also worth to mention that the paternal lineage of an early Neolithic Moroccan individual appeared to be distantly related to the typically North African E-M81 haplogroup^30^, suggesting again a NW African origin of E-M183. A local origin of E-M183 in NW Africa > 2200 ya is supported by our TMRCA estimates, which can be taken as 2,000–3,000, depending on the data, methods, and mutation rates used.

The TMRCA estimates of a certain haplogroup and its subbranches provide some constraints on the times of their origin and spread. Although our time estimates for E-M78 are slightly different depending on the mutation rate used, their confidence intervals overlap and the dates obtained are in agreement with those obtained by Trombetta *et al*.^13^ Regarding E-M183, as mentioned above, we cannot discard an expansion from the Near East and, if so, according to our time estimates, it could have been brought by the Islamic expansion on the 7th century, but definitely not with the Neolithic expansion, which appeared in NW Africa ~7400 BP and may have featured a strong Epipaleolithic persistence^31^. Moreover, such a recent appearance of E-M183 in NW Africa would fit with the patterns observed in the rest of the genome, where an extensive, male-biased Near Eastern admixture event is registered ~1300 ya, coincidental with the Arab expansion^20^. An alternative hypothesis would involve that E-M183 was originated somewhere in Northwest Africa and then spread through all the region. Our time estimates for the origin of this haplogroup overlap with the end of the third Punic War (146 BCE), when Carthage (in current Tunisia) was defeated and destroyed, which marked the beginning of Roman hegemony of the Mediterranean Sea. About 2,000 ya North Africa was one of the wealthiest Roman provinces and E-M183 may have experienced the resulting population growth.

The genetic landscape of North Africa has been strongly influenced by geography. Past and recent historical migrations gave rise to a complex genetic landscape. A recent study on genome-wide data has shown a lack of correlation between geographical and genetic populations^20^. However, when we turn to the Y chromosome, it has been reported that the Y-chromosomal variation is strongly structured within the region^22^. Interestingly, this strong geographical structure becomes somehow diluted when we go deeply into a particular Y chromosomal branch, which could be attributed to the rapid radiation mentioned above. E-M183 subbranches have spread through all the area, and are now represented in all the populations sampled. Despite this lack of structure within E-M183, a different pattern could still be appreciated in coastal populations when compared with more inland territories (Western Sahara, Algerian Reguibates). This pattern has been observed with genome wide data^20^ and could be related to migrations along the coast. And as mentioned above, principal component analysis suggested that PF6789 could be driving this different component. Figure 2 shows that PF6789 is very frequent in Oran (Algeria), Tunisia and Libya, and is present in the Near East and the Iberian Peninsula. Finally, surprisingly the highest frequency of M183* is shown in the Iberian sample which, given the low frequency values of E-M183 in the area, could be attributed to genetic drift acting on a low-frequency variant.

The present study has provided a phylogeographic refinement of the North African lineage E-M183. Time estimates based on whole Y chromosome data have been contrasted to those obtained by Y-STRs in order to provide an updated TMRCA for E-M183 carriers. Despite the analysis of E-M183 and its subbranches has shed some light into the historical and demographic processes that could have given rise to the North African genetic landscape, more efforts should be done, probably by incorporating whole genome data, to better understand the North African genetic landscape. And indeed, our study strongly showcases the need of incorporating ancient DNA in the study of paternal North African structure, in order to understand the genetic background of the area before the coalescence time of E-M183.

## Materials and Methods

### Samples/ethics

We sequenced fifteen males from seven North African populations (Morocco, Libya, Algeria, Western Sahara, Tunisia, Berber Zenata and Berber Tunisia) by using whole-genome shotgun paired-end sequencing (Illumina HiSeq 2000) to a mean coverage of 30x. North African individuals were selected for belonging to the E-M183 haplogroup by using TaqMan (Life Technologies) probes. DNA donors were recruited with informed consent. All experimental protocols were approved by the Institutional Review Board of the Comitè Ètic d’Investigació Clínica-Institut Municipal d’Assistència Sanitària (CEIC-IMAS) in Barcelona (2013/5429/I) and were carried out in accordance with the approved guidelines. Moreover, 21 E-M183 Y chromosome sequences were supplied by YFull, which is a Y Chromosome sequence interpretation service (http://www.yfull.com/). We also included 32 additional Y chromosome sequences belonging to different haplogroups in order to build a reliable phylogeny. Some of these sequences were obtained from published studies^32–35^, other sequences were also supplied by YFull and some sequences are still unpublished (Lorente-Galdós *et al*., unpublished work). Overall, the initial Y chromosome dataset consists of 68 individuals (see Supplementary Table S1).

In order to validate the new variants within the E-M183 branch discovered by whole Y chromosome sequences and to study its genetic diversity at a population level, we used a larger dataset that includes 454 males from North African, Iberian, and Near Eastern populations (Supplementary Table S4). Donor samples were collected with the appropriate informed consent.

### Data analysis, Variant Calling and Filtering

All sequence reads, both those obtained by whole-genome shotgun paired-end sequencing (Illumina HiSeq technology) and those supplied by other sources, were mapped to the human Y chromosome reference sequence (hg19/GRCh37) with BWA^36^. After that, we removed PCR duplicates, recalibrated base quality scores and performed an indel realignment applying the Genome Analysis Toolkit (GATK) v3.4–46^37^.

Variant calling was performed across the 68 initially selected samples simultaneously using the GATK UnifiedGenotyper tool^37^ leading to a raw call set of 107,672 SNPs. Then, we applied hard filtering parameters according to GATK best practices recommendations^38,39^ and ended up with 23,542 variants. After that, we considered only those sites supported by ≥5 reads, what we called the callable region, which reduced the dataset to 7,535 variant sites (see Supplementary Fig. S4).

Since the Y chromosome is especially rich in repeats, duplications and low-quality regions, we restricted our analysis to high quality regions defined by Wei *et al*.^8^, in order to avoid biases introduced by repeated sequences. After excluding ampliconic segments and heterochromatic, pseudoautosomal and X-transposed regions (8.97 Mb) from our dataset, we obtained 4.88 Mb of unique Y chromosomal sequence (<9% of the total Y chromosome length; see Supplementary Table S5 and Supplementary Fig. S4).

Finally, we used AWK in-house scripts to remove heterozygous calls, and VCFtools^40^ to discard those samples with ≥5% missing calls and/or a sequence read depth of <10X. At the end, our dataset contained 62 individuals and 4,269 variable sites.

### Phylogenetic inference and dating

Haplogroups were assigned using AMY-tree v1.2^41^. We considered an updated version of the Y chromosome haplogroup tree, as well as the Phylotree and ISOGG databases and the hg19 Y chromosome FASTA sequence (obtained from the UCSC genome browser, http://hgdownload.cse.ucsc.edu/goldenpath/hg19/chromosomes/). Results obtained are consistent with prior haplogroup assignation^32–35^ and, in the case of samples sequenced for this study, haplogroups are consistent with the experimental validation.

A FASTA formatted alignment with the high quality variable sites of all samples was built and used to produce a maximum parsimony tree using MEGA5^42^ (Fig. 1).

We calculated the TMRCA and its standard deviation for E-M81, as well as the divergence time from and its sister clade (E-M78), with two independent methods: i) a Bayesian estimation of the node ages performed with BEAST v1.8.2^43^. Markov chain Monte Carlo (MCMC) samples were based on 15,000,000 generations and the first 1,500,000 generations (10%) were discarded as burn-in. For the analysis, we combined the outputs of five independent runs using LogCombiner. In all runs, we used GTR as a substitution model under a strict clock. As a coalescent tree prior, we used an expansion model using the priors specified by Trombetta *et al*.^13^. Finally, in order to reduce the BEAST computational time, we included only variant sites and multiplied the substitution rate by the number of callable sites (4.88 Mb) and divided it by the number of variable sites (4,269 sites). And ii) the rho statistic^44^, as implemented in the Network 5.0 software (www.fluxus-engineering.com). This statistic is linearly related to time and mutation rate (ρ = μT), assuming rate constancy across the tree branches.

Given the uncertainty regarding the mutation rate, both for BEAST and rho estimates we used two different substation rates: i) a ‘fast’ rate^2^ of 10^−9^ substitutions/site/ year; and ii) a ‘slow’ rate^24^ of 6.17 × 10^−10^ substitutions/site/year.

For the analysis of the effective population size, Bayesian skyline plots were generated using BEAST v1.8.2^43^. Markov chain Monte Carlo (MCMC) samples were based on 15,000,000 generations, and the first 1,500,000 generations (10%) were discarded as burn-in. Again, five different runs were combined using Logcombiner and, in all of them, we used GTR as a substitution model under a strict clock and a linear piecewise linear skyline model with 10 groups. We used the same fast and slow rates as for the dating methods described above.

### Haplogroup frequencies and validation of new variants

An updated map of E-M81 haplogroup frequency distribution (Supplementary Fig. S1) was constructed using the Surfer Golden software v 10.0.500 (Golden Software, Golden, CO, USA), in which we included haplogroup frequencies obtained in this study, as well as frequencies obtained in other studies^45–60^. AWK in-house scripts have been used to define all variants within E-M183 (see Supplementary Fig. S5 and Supplementary Table S6). In particular, five of those variants are defining new subclades (SM001, PF6794, PF6789, CTS12227, Z5009) within the North African phylogenetic branch E-M183 (Fig. 1b, Supplementary Fig. S6). To study those subbranches at a population level, we validated them in a set of 454 North African, European, and Near Eastern individuals (see Methods; Supplementary Table S7). First, we selected samples with the derived allele at M183 by using single TaqMan (Life Technologies) probes. Overall, 250 individuals were derived for M183 and, subsequently, selected to validate the five new variants by Taqman (Life Technologies) assays by using the manufacturer’s protocols (Supplementary Table S8).

### Y-STR analysis

The individuals that were derived for E-M183 were subsequently typed for the 17 Y-STRs (DYS19, DYS385a/b, DYS389I, DYS389II, DYS390, DYS391, DYS392, DYS393, DYS438, DYS439, DYS437, DYS448, DYS456, DYS458, Y GATA H4, and DYS635) contained in the AmpFlSTR®YFiler® PCR Amplification kit (Life Technologies, Carlsbad, CA, USA)^61^ (Supplementary Table S9). However, the final dataset consists on 12 Y-STRs and 185 individuals to eliminate missing values. Diversity, average number of pairwise differences and heterozygosity were calculated using Arlequin 3.5^62^. In order to explore whether E-M183 (and its subbranches) showed any signal of population structure, a hierarchical analysis of molecular variance (AMOVA) based on Fst was performed using Arlequin 3.5^62^. We group the populations using two criteria: i) according to their geographical origin; and ii) according to their E-M183 subbranches. Median-joining networks were drawn with Network 5.0.0.1 (www.fluxus-engineering.com). Finally, the TMRCA has been estimated with the ρ approach, implemented within the program Network 5.0.0.1^63^, and using a mutation rate of one mutation per haplotype per 858 years, obtained from the compilation in the YHRD database (www.yhrd.org, accessed on Dec. 14th, 2016) for this set of 13 Y STRs and using a generation time of 30 years^64^.

### Data availability statement

The datasets generated and analysed during the current study are available in https://figshare.com/articles/North_African_Ychromosome_dataset_vcf/5537941.

## Electronic supplementary material

Supplementary_figuresS1-S6

Supplementary_tablesS1-S9
