# Supplementary material for: Whole Y-chromosome sequences reveal an extremely recent origin of the most common North African paternal lineage E-M183 (M81)
Source: Sci Rep. 2017 Nov 21;7:15941. doi: 10.1038/s41598-017-16271-y (PMC5698413; doi:10.1038/s41598-017-16271-y)

Whole Y-chromosome sequences reveal an extremely recent origin of the most common North African paternal lineage E-M183 (M81)

Neus Solé-Morata[1], Carla García-Fernández[1], Vadim Urasin[2], Asmahan Bekada[3], Karima Fadhlaoui-Zid[4], Pierre Zalloua[5], David Comas[1*], Francesc Calafell[1*]

1. Institute of Evolutionary Biology (CSIC-UPF), Departament de Ciències Experimentals i de la Salut, Universitat Pompeu Fabra, Barcelona, Spain.

2. YFull – Research Group, Russia.

3. Département de Biotechnologie, Faculté des Sciences de la Nature et de la Vie, Université Oran 1 (Ahmad Ben Bella), Oran, Algeria

4. Laboratoire de Génetique, Immunologie et Pathologies Humaines, Faculté des Sciences de Tunis, Campus Universitaire El Manar II, Université El Manar, Tunis, Tunisia

5. The Lebanese American University, Chouran, Beirut, Lebanon

Corresponding authors: Dr. David Comas and Dr. Francesc Calafell, Institut de Biologia Evolutiva (UPF-CSIC), Departament de Ciències Experimentals i de la Salut, Universitat Pompeu Fabra, 08003, Barcelona, Catalonia, Spain. Tel.: +34 933160842; E-mail address: david.comas@upf.edu, francesc.calafell@upf.edu

Supplementary Figure S1. Contour maps of the derived allele frequencies of EM81 constructed using the Surfer Golden software v14 (Golden Software, Golden, CO, USA) (http://www.goldensoftware.com/products/surfer)

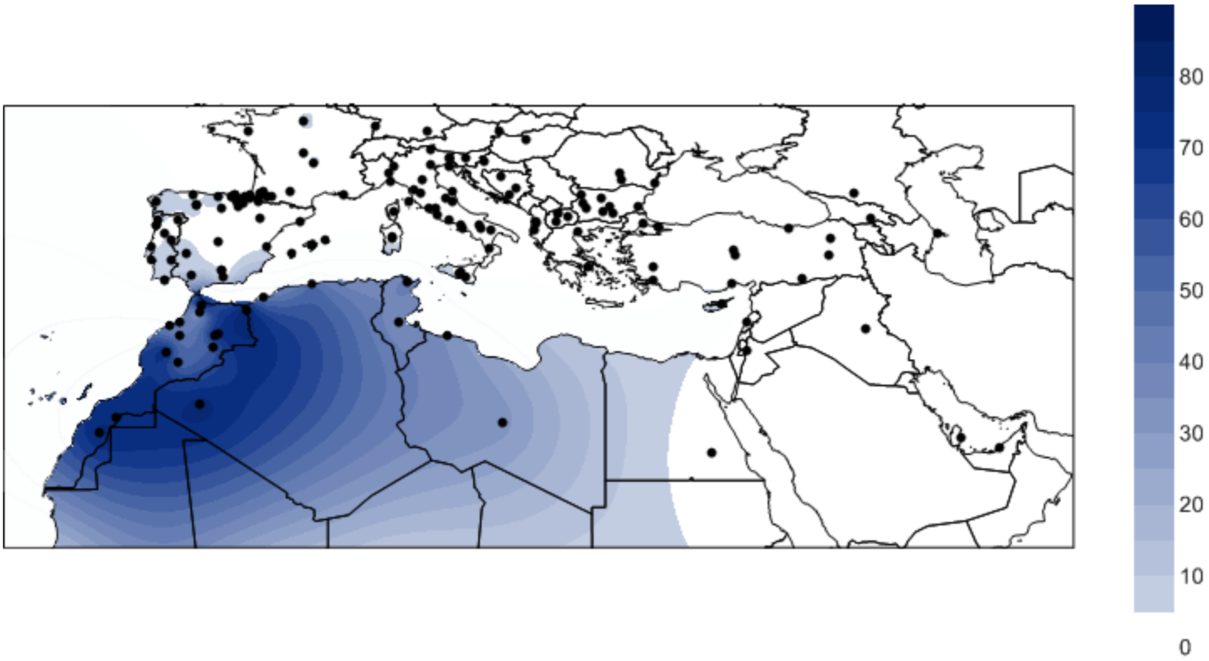

Supplementary Figure S2. Box plots of the mean values of PCA1 (a and c) and PCA2 (b and d). Populations are shown in a) and b), and haplogrups in c) and d). ALG_ORN, Oran (Algeria); ALG_RG, Reguibates (Algeria); IBS, Iberian Peninsula; LIB, Libya; MOR, Morocco; ME, Middle East; SAH, Western Sahara; TUN; Tunisia.

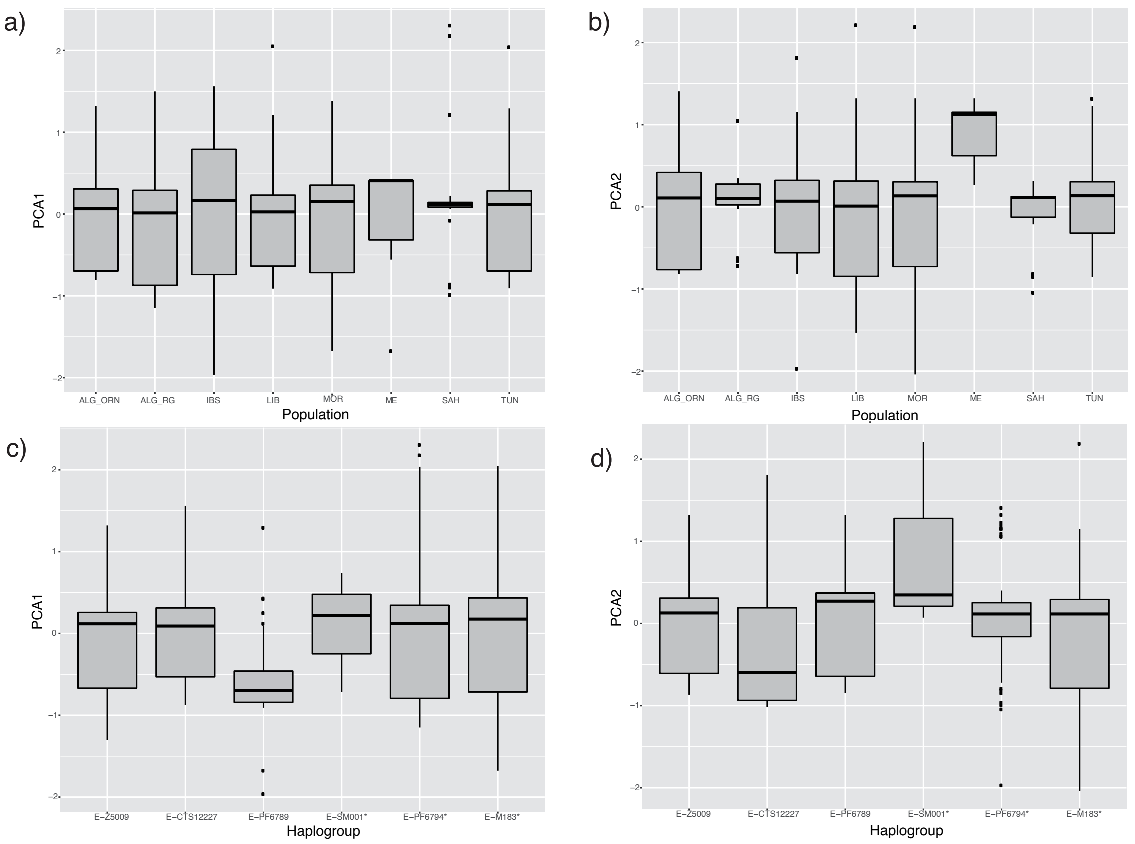

Supplementary Figure S3. Diversity indices as a function of the longitude. Population a) gene diversity, b) heterozygosity and c) Y-STR haplotype variance in North Africa. Black lines are showing linear regressions of each diversity index onto longitude.

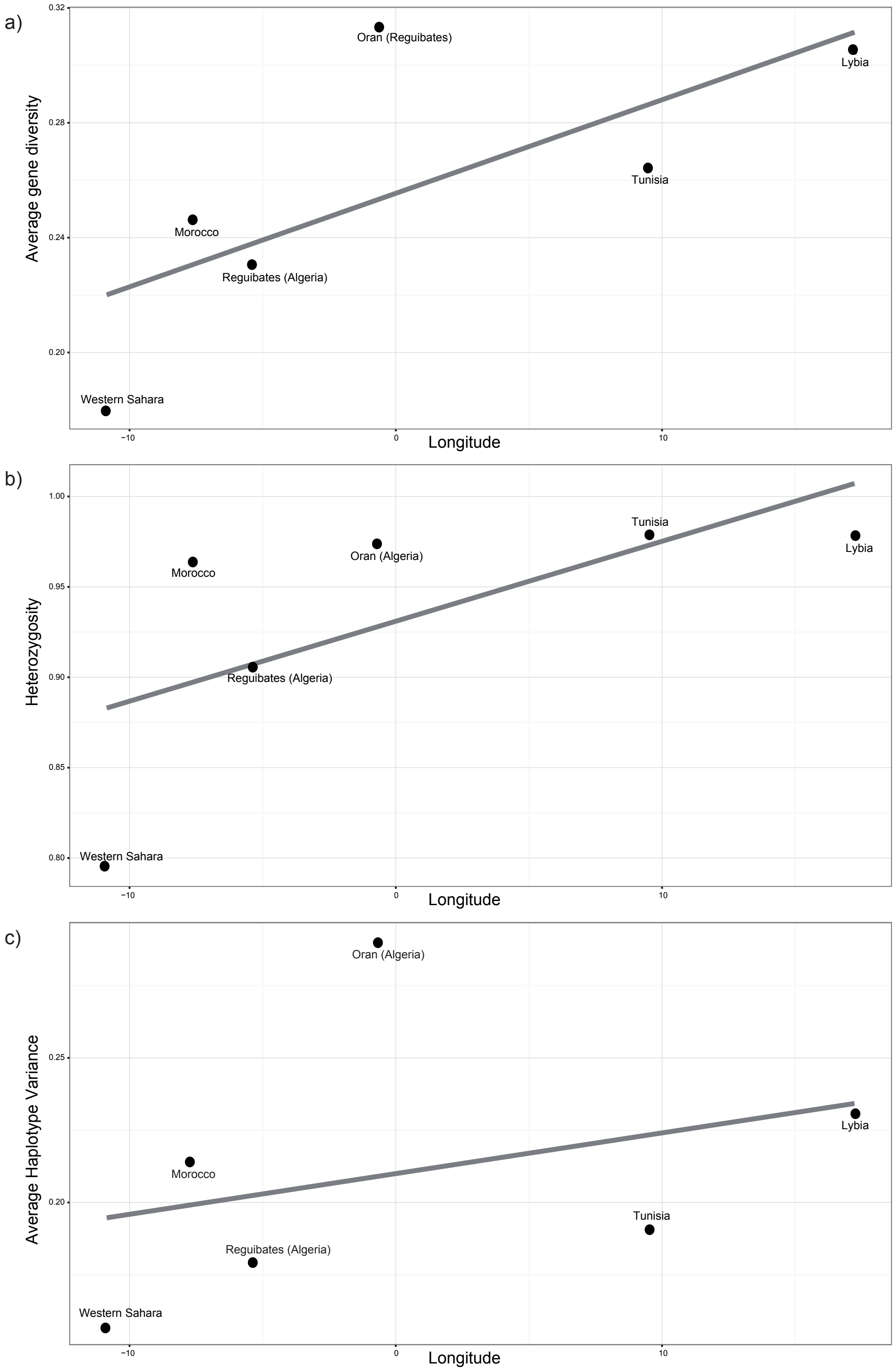

Supplementary Figure S4. Callability mask for the Y chromosome. Blue lines define the so-called unique regions defined by [8], vertical red lines define the callable region, and black lines indicate the regions that have been excluded for the analysis. Each black dot shows geographic data points where data was available.

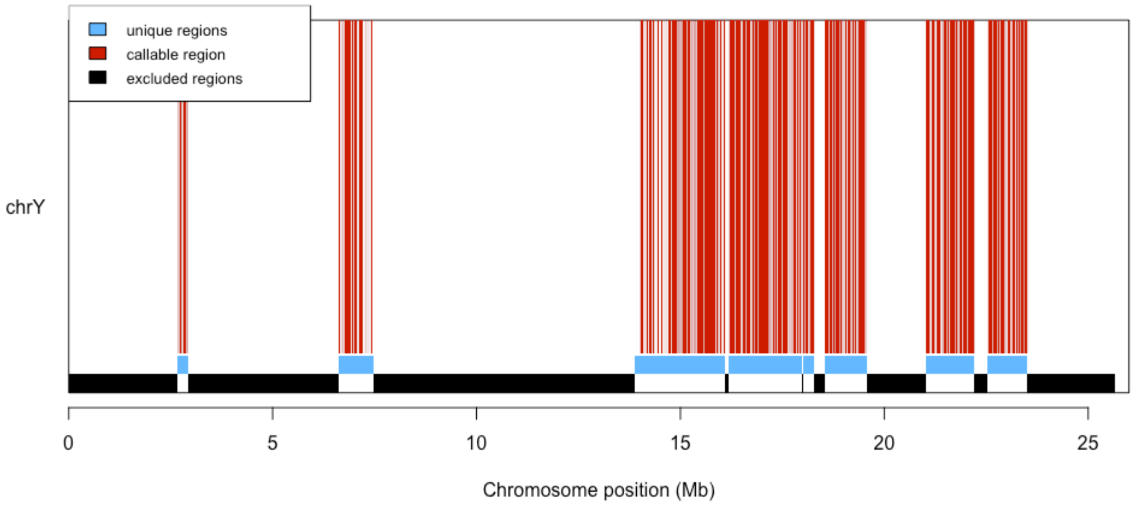

Supplementary Figure S5. Phylogenetic tree with numbered branches. To find variants on each branch see Supplementary Table S6.

```
            +ALG04
            |
         +--9-ALG05
         |  |
         |  +BZEN02
         |  |
         |  +SAU01
         |  |
         |  |  +BTUN01
         |  +-10
         |     +BTUN02
         |
         |  +SAH01
         |  |
         |  |  +MOR02
      +--8-11-12
      |  |  |  +MOR04
      |  |  |
      |  |  |  +FRE02
      |  |  +-13
      |  |  |  +PAL01
      |  |  |
      |  |  +LIB05
      |  |
      |  +SAH02
      |  |
      |  +BZEN01
      |  |
      |  |  +SAH03
      |  +-14
      |  |  +SAH04
      |  |
      |  |  +UK01
      |  +-15
      |  |  +USA02
      |  |
      |  +ALG03
      |  |
   +--7  |     +LIB02
   |  |  |     |
   |  |  |  +-17-TUN01
   |  |  |  |  |
   |  |  |  |  +LIB03
   |  |  |  |  |
   |  |  +-16  +LIB04
   |  |  |  |  |
   |  |  |  |  +MOR01
   |  |  |  |
   |  |  |  +MOR03
   |  |  |
   |  |  +ALG01
   |  |  |
   |  |  +FRE03
   |  |  |
   |  |  +USA01
+--6  |  |
|  |  |  +UK02
|  |  |  |
|  |  |  +TUN02
|  |  |  |
|  |  |  +ALG02
|  |  |
```

```
             |  |  +LEB01
             |  |
             |  |     +BUL02
             |  |     |
             |  |     |  +BUL03
             |  |  +-19-20
          +--5  |  |  |  +NOR02
          |  |  |  |  |
          |  |  |  |  +BUL01
          |  |  +-18  |
          |  |     |  |  +BEL01
          |  |     |  +-21
          |  |     |     +NOR01
          |  |     |
          |  |     +MAN02
          |  |
          |  |     +BAN03
       +--4  |  +-23
       |  |  |  |  +MBU01
       |  |  |  |
       |  |  +-22-YOR02
       |  |     |
       |  |     +YOR03
       |  |     |
    +--3  |     +YOR01
    |  |  |     |
    |  |  |     +EGY02
    |  |  |
    |  |  +-MAN01
    |  |
    |  |  +-DNK02
    |  +-24
    |     +-MBU02
    |
    |           +TOU
+---2        +-28
|   |        |  |  +EGY01
|   |     +-27  +-29
|   |     |  |     +LIB01
|   |     |  |
|   |     |  +-IRQ02
|   |     |
|   |  +-26-BAS01
|   |  |  |
|   |  |  |  +-HAN
|   |  |  +-30
|   +-25     +-DAI
|      |
|      |     +FRE01
|      |  +-32
|      +-31  +SAR
|         |
|         +--IRQ01
|
|  +--DNK07
1-33
|  |  +SAN04
|  +-34
|     +SAN02
|
+------SAN01
```

Supplementary Figure S6. Tree showing the phylogenetic positions of the five SNPs on a tree combining other reported SNPs. In red: SNPs genotyped for this study.

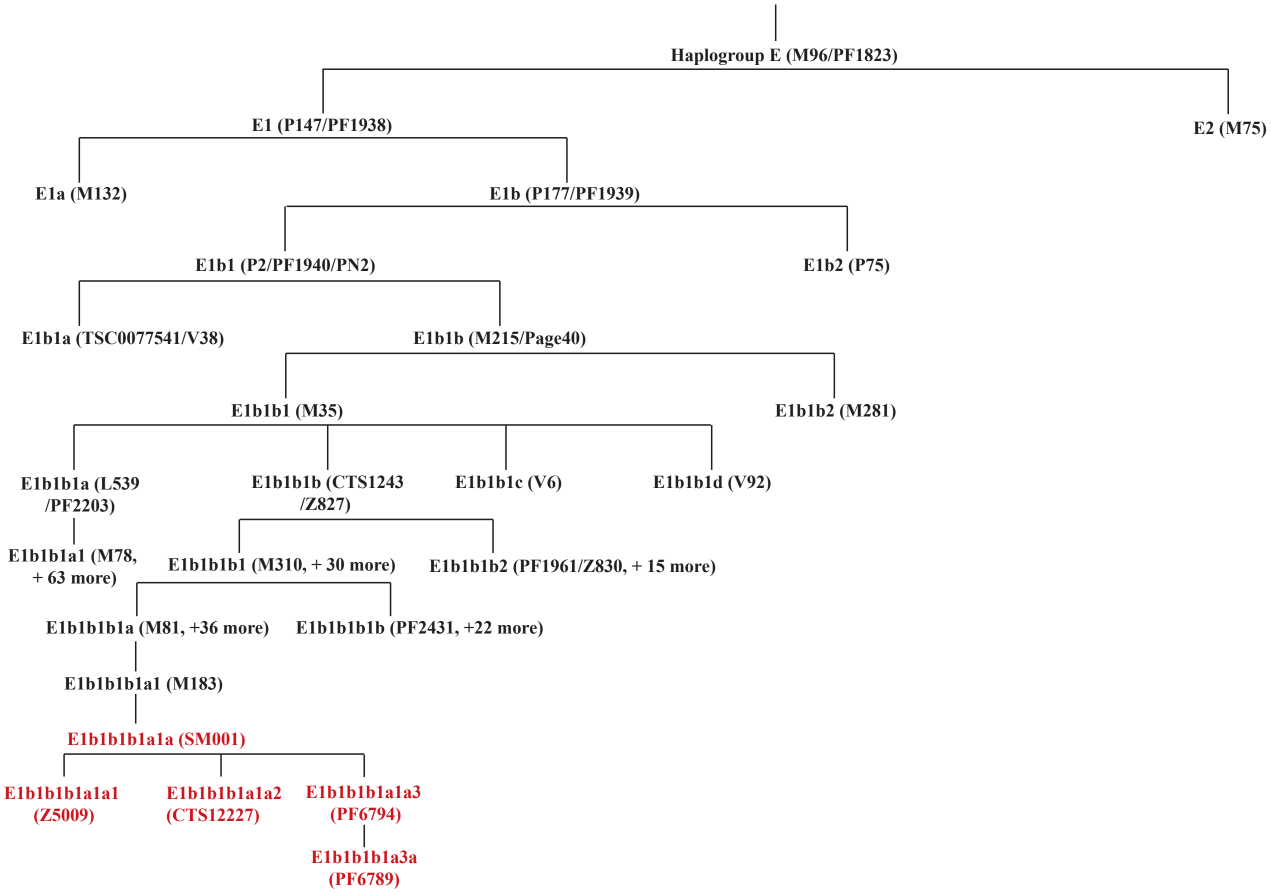

Supplement: Supplementary file 1 — Supplementary_figuresS1-S6 [file 41598_2017_16271_MOESM1_ESM.pdf]
